# A cluster randomized trial to measure the impact on nonsteroidal anti-inflammatory drug and proton pump inhibitor prescribing in Italy of distributing cost-free paracetamol to osteoarthritic patients

**DOI:** 10.1186/s12875-019-1050-4

**Published:** 2019-12-06

**Authors:** Massimo Vicentini, Pamela Mancuso, Paolo Giorgi Rossi, Sara Di Pede, Morena Pellati, Alberto Gandolfi, Daniela Zoboli, Daniela Riccò, Corrado Busani, Alessandra Ferretti

**Affiliations:** 1 Epidemiology Unit, Local Health Authority AUSL-IRCCS, Reggio Emilia, Italy , Reggio Emilia, Italy; 2 Pharmaceutical Department, Local Health Authority AUSL-IRCCS, Reggio Emilia, Italy; 3 Primary Health Care, Local Health Authority AUSL-IRCCS, Reggio Emilia, Italy; 4 General Practitioner, Local Health Authority AUSL-IRCCS, Reggio Emilia, Italy, Reggio Emilia, Italy; 5 Medical Directorate, Local Health Authority AUSL-IRCCS, Reggio Emilia, Italy, Reggio Emilia, Italy

**Keywords:** Paracetamol, Osteoarthritis, Proton pump inhibitors, Opioids, Nonsteroidal anti-inflammatory drugs, Drug prescription

## Abstract

**Abstract:**

**Background:**

Paracetamol is recommended as first-line treatment for pain control in osteoarthritis because it has fewer side effects than do other therapeutic options, including nonsteroidal anti-inflammatory drugs (NSAIDs). Prescribing proton pump inhibitors (PPIs) as gastric bleeding prophylaxis in chronic NSAID users is also common, although not recommended. In Italy, paracetamol is not reimbursed by the National Health System. The aim of this trial was to test whether the availability to osteoarthritis patients of free paracetamol would decrease their use of NSAIDs and, as a secondary objective, whether opioid and PPI consumption would also decrease.

**Methods:**

Eight general practitioners (GPs) (59 patients) were randomized to usual care and 8 (58 patients) to the experimental arm, where prescribed paracetamol was directly distributed for free by the local hospital. After 6 months, paracetamol was also available for free in the control arm.

The main outcome was the pre/post difference in average NSAID and PPI consumption. Differences between experimental and control arms in pre/post differences are reported, as registered by the drug prescription information system.

**Results:**

Average NSAID consumption decreased non-significantly, from 6.79 to 2.16 defined daily dose (DDD) in the experimental arm and from 3.19 to 2.97 DDD in the control group (*p* = 0.067). No changes were observed for PPIs (from 11.27 to 14.65 DDD and from 9.74 to 12.58 DDD in experimental and control arms, respectively, *p* = 0.788) or opioids (from 1.61 to 1.14 DDD and from 1.41 to 1.56 DDD in experimental and control arms, respectively, *p* = 0.419). When the intervention was extended to the control arm, no decrease in NSAID consumption was observed (from 2.46 to 2.43 DDD, *p* = 0.521).

**Conclusions:**

Removing small economic barriers had small or no effect on the appropriateness of opioid or PPI prescribing to patients with osteoarthritis; a reduction in NSAID consumption cannot be ruled out.

**Trial registration number:**

NCT02691754 (Approved February 24, 2016).

## Background

Osteoarthritis is the most common rheumatic disease; the Global Burden Of Disease estimates that about 26% of the Western Europe population over 70 years has pain or disability due to osteoarthritis [1].

Despite doubts on the effectiveness of paracetamol in the treatment of pain in osteoarthritis described in a recent meta-analysis, [2] many European and American guidelines recommend its use at high dose, (3 g/day) as a first-line drug for pain control because it is safer than other first-line options [3–6]. Paracetamol should be taken at regular intervals to be effective. Nonsteroidal anti-inflammatory drugs (NSAIDs) are also effective for pain control in osteoarthritis, with both analgesic and anti-inflammatory action, but they have adverse gastric, renal, and cardiac effects, particularly in older patients who are at high risk of NSAID-induced gastroduodenal lesions [7–9]. For this reason, most guidelines recommend NSAIDs only as second-line treatment, alone or in association with paracetamol [3, 5, 6]. Furthermore, NSAIDs are often prescribed in association with gastroprotective drugs, in particular proton pump inhibitors (PPIs) even if prophylactic use of PPI is not recommended [3].

Finally, opioids are recommended only as third-line treatments, when paracetamol and NSAIDs have failed or can no longer be tolerated. These drugs, however, also have serious side effects, such as constipation, nausea, and sedation. Furthermore, the most common commercial associations in Italy of opioid (codeine) and paracetamol do not allow the optimal dose of the opioid to be reached before reaching toxic doses of paracetamol.

In Italy, paracetamol is an over-the-counter drug, meaning that a GP’s prescription is not needed and that the cost to the individual is out of pocket. It is dispensed for free only through NHS hospitals or outpatient clinics. Three reasons have probably led to this unusual situation for an effective drug included in many guidelines as first-line treatment for several diseases: its very low price, that it can be bought without a prescription at any pharmacy, and the absence of any commercial interest in including it the list of drugs that can be prescribed through NHS by GPs.

As a consequence, paracetamol is only rarely prescribed by GPs as a first-line drug for osteoarthritis because the patient would have to pay for it entirely out of pocket [10]. Our hypothesis was that because paracetamol is not among the reimbursable drugs, NSAID prescription is more likely.

Many studies have showed the effect of reimbursement policies on drug prescriptions and consumptions [11, 12]; most of the studies included in two recent systematic reviews looked at the effect of restrictions on reimbursement, [12] cap payment, or co-payment strategies [11]. Only a few studies have observed the effect of relaxation or exemption from restriction strategies to induce the use of appropriate and effective drugs that may be underprescribed or under-used [13–17].

Therefore, we designed a trial to test whether giving GPs the possibility to prescribe to their osteoarthritic patients paracetamol dispensed by the NHS for free would decrease the use of NSAIDs and, as a secondary objective, whether opioid and PPI consumption would also decrease.

## Methods

### Setting

The trial was conducted in two primary care practices (composed of 8–10 GPs each, covering an area with a total of about 34,000 inhabitants) in the province of Reggio Emilia, in northern Italy. The 16 GPs working at the two practices each have 1500 patients, about one-quarter of whom are over age 65. The resident population can choose any GP in the province based on preference, though most residents choose their GP based on geographical convenience. The study was conducted from November 1, 2012 to October 31, 2013 and was subsequently divided in two main seasons: Winter-Spring (from November 1 to April 30) and Summer-Fall (from May 1 to October 31).

### Study design

All the GPs in the two practices were asked to participate in the trial. For the Winter-Spring season 2012–2013 (randomization period), those who agreed were randomized to one of the two arms: 1) usual prescribing practice and education about pain control in osteoarthritis; 2) new prescribing modality, i.e. free paracetamol and education about pain control in osteoarthritis. After 6 months, in the Summer-Fall season 2013, (implementation period), the control group also received prescriptions for free paracetamol. Randomization was performed centrally by the Reggio Emilia Epidemiology Unit using a generator of random numbers.

**Fig. 1:**
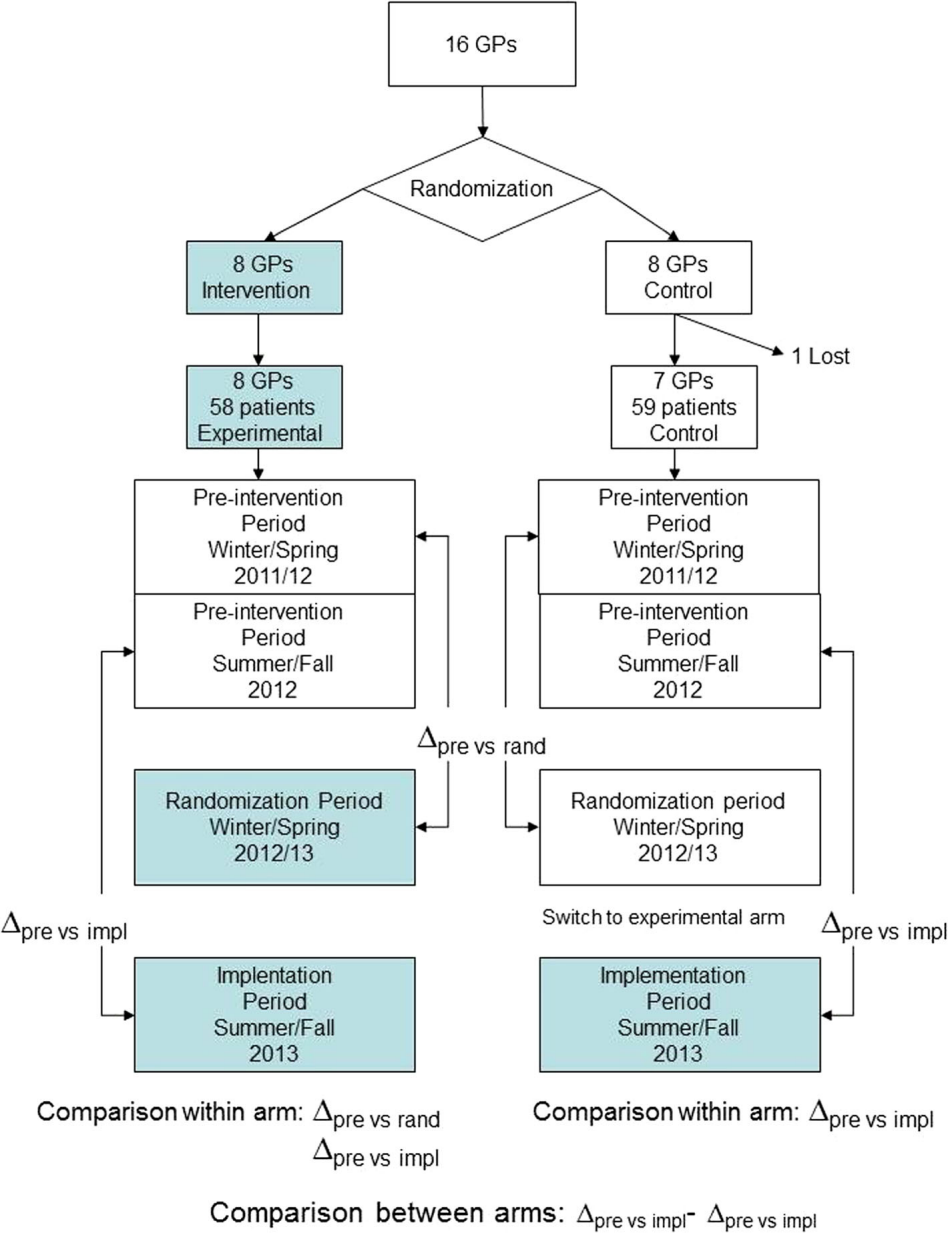
Flowchart of randomization process and comparisons between and within arms, by period

The principal endpoint was the difference in NSAID and PPI consumption in patients with osteoarthritis in the randomization period compared to the pre-intervention period (November 1, 2011 to October 31, 2012). As a secondary endpoint we also monitored the prescription of opioids. Differences in defined daily dose (DDD) per patient in the control and in the experimental arms were compared (Fig. 1).

### Outcome definition

The defined dose (DD) is the total sum of grams of paracetamol prescribed to each subject with osteoarthritis. The defined daily dose (DDD) represents the total sum of grams of paracetamol prescribed to each subject with osteoarthritis divided by 180 days (6 months, single period of observation). For the main endpoint the denominator also included patients who did not use that drug at all. We also present data on the proportion of users for each drug in the two periods.

The main comparison is the difference between the control and experimental arms in differences between pre-intervention and randomization period. We also report the comparison within each arm between the pre-intervention period and the randomization period and the comparison within the experimental arm for the pre-intervention and implementation period.

### Sample size

It was estimated that there were about 60 patients with osteoarthritis per GP (20% of the over-65-year-olds), 50 of whom are in active treatment. Further, it was assumed that about one-third (16 patients) were taking NSAIDs, with an average of 5 DDD per month and a standard deviation of 2.5. Setting alpha at 0.05, and assuming a design effect of clustering of 2, i.e. we doubled the sample size compared to a simple random sample, we estimated 90% power to detect the relevant difference of halving NSAID consumption randomizing at least 8 GPs per arm; we had the opportunity to randomize 16 GPs.

### Participants and randomization procedure

All the 16 GPs working in the two primary care practices were asked to participate in the study. All agreed to participate and signed an informed consent. The randomization unit was the GPs, while the statistical units were osteoarthritic patients. The GPs were randomized centrally by the Epidemiology Unit with a pseudo-random number generator using the first number of most recent extraction of the National Lottery as the seed. The allocation of the arm was also randomly assigned to the two groups.

### Description of the intervention

All the participating GPs received a short course on pain control in patients with osteoarthritis.

The GPs in the experimental arm were also briefly informed of the opportunity to prescribe free paracetamol; the patients go to the local hospital or public clinic with the GP’s prescription to receive the paracetamol.

### Data sources

The data on drug consumption were taken from the pharmaceutical information system. The information system merges two databases, one recording all the drugs charged to the Italian NHS by pharmacies and one recording all the drugs directly administered by hospitals and outpatient clinics. The system collects information on the patient, the prescriber, the dose, and the drug. It does not include drugs purchased out of pocket at pharmacies.

All the GPs provided a list of their patients with osteoarthritis seen during the study period (November 1, 2012 to October 31, 2013). Patients who did not gave consent for data management and treatment to the GP, would be automatically excluded from the lists. We are not aware of any case of exclusion.

At the end of the study, all the GPs filled in an ad hoc closed-ended questionnaire on the feasibility and acceptability of the new prescription modalities. Questions included GP and patient satisfaction, proportion of patients complying with the proposed protocol at the beginning and at the end of the period, and an open-ended question for comments (see Additional file 1: questionnaire for GP).

### Data analyses

The lists of patients provided by the GPs were used to define the population of patients with osteoarthritis.

All data are presented with 95% confidence intervals, built taking into account the cluster randomization using a random effect model to estimate the correct variance. To take into account clustering on GP, all the analyses were conducted with the statistical package for complex survey data of STATA 13.0. Differences within and between arms (Winter-Spring 2011–12 vs. Winter-Spring 2012–13 and Summer-Fall 2012 vs Summer-Fall 2013) were tested using linear regression model adjusting by sex and age. We present the design effect for baseline drug prescription among GPs and for the comparison between experimental and control arm.

In order to prevent seasonal fluctuations and to have the same probability of at least one prescription event, we compared the same time lapse in the pre-intervention period and in the randomization period, i.e. for the main objective analysis, we compared Winter-Spring 2011–12 (pre-intervention period) with the Winter-Spring 2012–13 (randomization period) in the two groups. Differences between pre- and post-intervention periods in the intervention and control arms were compared with a two-tailed t-test; *p*-value < 0.05 was considered as threshold to reject the null hypothesis.

In order to confirm the trial results, we also compared the changes in the control arm between the pre-intervention period (Summer-Fall 2012) and the last 6 months of the study (Summer-Fall 2013, implementation period) during which the control arm could also receive paracetamol for free with the new prescribing modality.

## Results

Of the approximately 20,000 patients, the GPs identified 117 with osteoarthritis that required a visit or a prescription during the study period: 58 in the experimental arm and 59 in the control arm. One GP, in the control arm, stated he had not seen any patient with osteoarthritis during the study period. In the control arm the mean of treated patients was 8 per GP (range: 4–17); in the intervention arm, the mean was 7 patients per GP (range: 1–20) (Table 1). At baseline, more DDD were prescribed in the experimental arm than in the control arm. The variance due to GP clustering was small (design effect 1.12, 1, and 1, for NSAIDs, opioids, and PPI, respectively).

**Table 1:** Descriptive characteristic of osteoarthritic patients and General Practitioners and Defined Daily Dose (DDD) during the year preceding the study period

	Experimental arm	Control arm
	N (%)	N (%)
General Practitioners (GPs)	8	7
Sex		
F	1 (12.5)	3 (42.9)
M	7 (87.5)	4 (57.1)
Number of patients		
Mean	7.3	8.4
Range	(1–20)	(4–17)
Patients	58	59
Sex		
F	46 (79.3)	49 (83.1)
M	12 (20.7)	10 (16.9)
Age		
Mean	79.6	78.5
Range	(65–95)	(65–97)
Age classes		
65–74	15 (25.9)	16 (27.1)
75–84	27 (46.6)	30 (50.8)
85+	16 (27.6)	13 (22.0)
Prescription during the year before randomization		
NSAIDs		
Users	27 (46.6)	28 (47.5)
Total DDD in the period	2328.7	1030.6
*DDD per patient, median (IQR)*	40 (20–80)	26.5 (13.9–60)
Average DDD per day	6.38	2.82
*DDD per day per patient, median (IQR)*	0.11 (0.05–0.22)	0.07 (0.04–0.16)
Opioids		
Users	16 (27.6)	22 (37.3)
Total DDD in the period	627.0	521.4
*DDD per patient, median (IQR)*	14.5 (4–37.2)	8 (4–20)
Average DDD per day	1.72	1.43
*DDD per day per patient, median (IQR)*	0.04 (0.01–0.10)	0.02 (0.01–0.05)
PPIs		
Users	28 (48.3)	27 (45.8)
Total DDD in the period	4249.0	3719.3
*DDD per patient, median (IQR)*	119 (28–203)	133 (42–182)
Average DDD per day	11.64	10.19
*DDD per day per patient, median (IQR)*	0.33 (0.08–0.56)	0.36 (0.12–0.50)

*PPIs* Proton Pump Inhibitors, *NSAIDs* Non-steroidal Anti-Inflammatory Drugs, *DDD* Defined Daily Dose, *IQR* interquartile range

The comparison of the pre-intervention period (Winter-Spring 2011–12) with the randomization period (Winter-Spring 2012–13) in the control arm showed an average increase of 0.048 DDD in PPI consumption [95% CI: − 0.01; 0.11] but no trend for NSAIDs or for opioids. An average increase 0.058 in PPI consumption was observed in the experimental arm [95% CI: − 0.01; 0.13], while a variation of − 0.08 DDD in NSAID consumption [95%IC: − 0.16; 0.00] and a variation of − 0.008 DDD in opioids [95% CI: − 0.03; 0.02] were observed.

The experimental arm had a higher consumption of NSAIDs than did the controls in the pre-intervention period; after the intervention, the consumption in the experimental arm was very similar to that of the control arm. Thus, the analysis of the difference in differences (Table 2, between arms comparison column) gave a borderline statistically non-significant reduction of NSAIDs. with − 0.081 DDD [95% CI: − 0.17; 0.01]. For PPIs and opioids, the intervention showed no effect, with a minimal increase for the first – 0.013 [95% CI: − 0.08; 0.11] – and a small decrease for the second – -0.012 [95% CI: − 0.04; 0.02]. The variance between GPs in the effect size was negligible (design effect 1 for all the comparisons between arms).

**Table 2:**
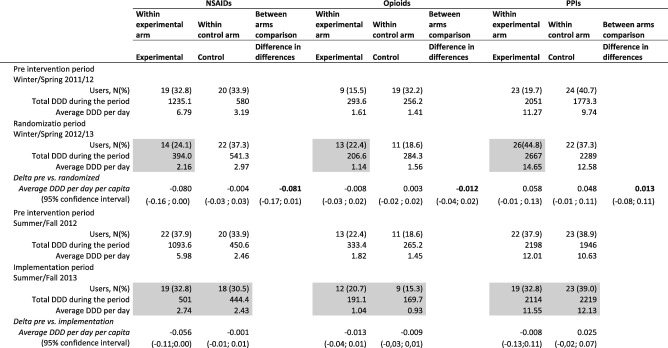
Defined Daily Dose (DDD) and Defined Dose (DD) per patient variation for Non-steroidal Anti-Inflammatory Drugs (NSAIDs), Proton Pump Inhibitors (PPI) and opioids by arm and period

*Gray parts* intervention, free paracetamol. In bold the main comparisons of the trial, i.e. the difference between intervention and control pre and post delta. *PPIs* Proton Pump Inhibitors; *NSAIDs* Non-steroidal Anti-Inflammatory Drugs; *DDD* Defined Daily Dose; DDD per capita

The comparison of the pre-intervention period (Summer-Fall 2012) with the last 6 months of the study (Summer-Fall 2013), when the control group shifted to the new prescribing modality, showed only a small non-significant reduction in the consumption of NSAIDs.

During the intervention, 53 of the 58 (91%) osteoarthritic patients in the experimental arm went to the hospital to pick up the paracetamol. The number of NSAID users did not substantially change in either arm. For example, during the pre-intervention period there were 19 NSAID users (33%) in the experimental arm, decreasing to 14 (24%) during the intervention; in the control arm the number of users increased from 20 (34%) to 22 (37%) in the same two periods. The differences for opioids and PPIs showed an opposite trend: when comparing the pre-intervention with the randomization period, the proportion of users decreased in the control arm and increased in the experimental arm.

In the control arm during the implementation period, when free paracetamol was offered to all the participants, 30 subjects (51%) went to the hospital to pick up the drug. The number of NSAID users was similar in the two periods: 20 (34%) in the pre-intervention period, and 18 (31%) when the free paracetamol was offered. The number of users for opioids and PPIs did not change over the two periods.

### Acceptability

The GPs appreciated the new prescribing modality (73% good or optimal satisfaction), reporting similar satisfaction of their patients (67% good or optimal); very few patients were surprised by or disappointed about having to pick up the drug at the hospital, although about one-third did not continue to do so throughout the entire study period. In the control arm of 59 potential users of paracetamol, 30 actually picked up the drug (50%); in the experimental arm, 53 of 58 patients picked up the drug.

## Discussion

### Efficacy

Our study showed small or no effect of offering the chance to prescribe paracetamol for free on reducing NSAID consumption in patients with osteoarthritis. This effect, with borderline statistical significance, was not reproducible when we expanded the intervention to the control arm. It must be noted that the experimental arm had a higher level of NSAID consumption and that after the intervention consumption reached almost the same level as pre-intervention in the control arm. The effect in the experimental arm was mostly due to the shift of a few heavy users from NSAIDs to paracetamol. Furthermore, only half of the patients in the control arm actually picked up the paracetamol at the hospital when the new prescribing modality was made available to them. Finally, it must be noted that all the GPs received an education and training course on drug use for pain control just before the start of recruitment. The simultaneity of the training and the new way of delivering the paracetamol probably led to a more positive attitude in the experimental arm than did the introduction of the new strategy 6 months after the training course, as occurred in the control arm. We can exclude that the lack of effect in the second period was influenced by the evidence reported in the recently published systematic review [2] about better efficacy of NSAIDs compared to paracetamol because most of the trials included were already available before 2013 and did not influence the GPs’ attitude during the first phase of the study.

Although the research question of this trial was contextualized in circumstances that are quite specific to Italy, how to change prescribing habits and how even small economic barriers can influence these habits are much more universal issues.

Only a few studies have showed effective interventions to modify prescribing habits [18, 19], in particular for NSAIDs [20]. A few other studies have tried to evaluate the efficacy of removing economic barriers to appropriate drug prescribing, but not specifically regarding paracetamol [12, 21]. Most of these studies have been interested in increasing the appropriate use of drugs considered to be underprescribed in order to increase the health benefits. In our study, a shift from NSAIDs to paracetamol cannot reasonably lead to an immediate health gain; the main advantage of paracetamol is its safety profile, particularly in terms of gastric complications, which can easily be prevented with PPIs. Therefore, our study could not aim to detect a decrease in the number of complications but only to try to record the reduction of NSAID prescriptions. Our interest is in decreasing the chronic use of NSAIDs because of this vicious circle of prescriptions, possible complications, and prescription of prophylactic PPIs, which do not increase health benefits but increase the risk of long-term complications, costs, and the complexity of patient management.

However, we did not observe any decrease in PPI prescriptions. Many authors have suggested that one of the main drivers of PPI prescription is its prophylactic use in association with NSAIDs [22, 23]. We do not know whether this is also the case for osteoarthritic patients, but the observation that when the use of NSAIDs was reduced by half, PPIs did not show a corresponding decrease, suggests that there are other reasons for prescribing them. Nevertheless, changing the prescribing habits for PPIs proved to be a very difficult task [24–26] and only complex interventions or large-scale policies have been shown to be quite effective [26, 27].

Opioid prescribing, as expected, did not change during the study period. The intervention was not expected to modify prescribing of second- or third-line drugs [23]; this secondary endpoint was only to monitor any possible indirect effects.

### Acceptability and feasibility

The new prescribing modality was well accepted by the GPs, who also declared that very few patients claimed that it was inconvenient to pick up the paracetamol directly at the hospital. On the other hand, the GPs declared that about one-third of the patients did not continue the treatment for the entire study period, which was quite short for a chronic disease, i.e. 6–12 months.

The dispensing of paracetamol directly by hospital is in line with Italian legislation, even though the prescription by GPs was experimental. While direct dispensing in local hospital and public clinics was the only viable solution for prescribing paracetamol for free, it may have been more time consuming than is purchasing it at the pharmacy. The effectiveness of the intervention may have been reduced because while we did remove a small economic barrier, we could not remove logistical ones.

The workload for the hospital pharmaceutical service was relatively small, even in the hypothesis of scaling up the distribution to the whole population of the district.

### Limits and strengths

Unfortunately, we do not have any information on out-of-pocket drug consumption; given that paracetamol and many NSAIDs are not expensive, we have only a partial picture of the drug consumption. It must be noted that the average NSAID DDD/per patient is extremely low, suggesting an important out-of-pocket use. The same may apply for some patients that followed the recommendation of shifting to paracetamol, purchasing it at a pharmacy rather than going to the hospital. Furthermore, we do not have any information on the quantity of paracetamol distributed at the hospital, only on the number of patients that took advantage of this opportunity.

The study reached a smaller sample size than was planned because we overestimated the number of patients receiving drug prescription per GP and the average use of NSAIDs per patient. This, however, was partially counterbalanced by a smaller intra-cluster correlation as the design effect was only 1.1 instead of the 2 we predicted. Unfortunately, what really decreased the power of our study was a worse than expected standard deviation/mean ratio: we predicted a 0.5 ratio but observed almost a ratio of 2. As a consequence, the study was underpowered, and even halving the prescribed NSAID, DDD resulted statistically non-significant.

We adopted a study design with a main comparison between arms with a difference in differences approach. Furthermore, we had the opportunity to check whether the advantages observed in the comparison between arms was also achievable when we extended the intervention to the control arm. If we only look at the analyses on the main comparison, we could conclude that the intervention was effective in reducing NSAIDs and not effective in reducing opioids or PPIs. Looking also at the confirmatory analyses, however, we have more doubts as to the effectiveness of the intervention, at least when it is implemented in practices that have a low baseline per capita consumption of NSAIDs.

## Conclusions

In this study we tested the effectiveness of removing small economic barriers on the prescribing appropriateness of pain control drugs in patients with osteoarthritis. Our data showed an effect, although non-statistically significant, only for the reduction of NSAIDs but not of opioids or PPIs. Furthermore, when the intervention was extended to the control arm, the results were not reproduced. The need for multifaceted interventions when trying to modify GPs’ prescribing habits and patients’ drug consumption has been already pointed out [28–30]; our study suggests that administrative intervention alone has a limited effect, as does training alone.

It is important to continue conducting research on factors influencing drug prescribing behaviours to design possible effective interventions to improve prescribing appropriateness.

## Supplementary information

**Additional file 1.** questionnaire for GP.

## Data Availability

The authors can provide anonymous micro-data on request to all the researcher who make a request reporting the objective of the re-analyses and a statistical analysis plan, after approval of the Area Vasta Emilia Nord Ethic Committee.

## Abbreviations

DD: Defined dose
DDD: Defined daily dose
GP: General Practitioner
NHS: National Health System
NSAIDs: Nonsteroidal anti-inflammatory drugs
PPIs: Proton pump inhibitors

## References

[1] Institute for Health Metrics and Evaluation (IHME). GBD Compare Data Visualization. Seattle, WA: IHME, University of Washington, 2018. Available from http://vizhub.healthdata.org/gbd-compare. (Accessed Oct 2019).

[2] da Costa BR, Reichenbach S, Keller N, Nartey L, Wandel S, Jüni P, Trelle S (2016). Effectiveness of non-steroidal anti-inflammatory drugs for the treatment of pain in knee and hip osteoarthritis: a network meta-analysis. Lancet.

[3] NICE clinical guideline 59 Osteoarthritis: the care and management of osteoarthritis in adults. Febbraio 2008. Disponibile al sito: http://www.nice.org.uk/CG059 (ultimo accesso 30/Sept/2010).

[4] Chou R, Fanciullo GJ, Fine PG, Adler JA, Ballantyne JC, Davies P (2009). Clinical guidelines for the use of chronic opioid therapy in chronic non-cancer pain. J Pain.

[5] ICSI Health Care Guideline (2009). Assessment and Management of Chronic Pain.

[6] American Geriatric Society (2009). Pharmacological management of persistent pain in older persons. J Am Geriatr Soc.

[7] Farrar JT, Young JP, LaMoreaux L, Werth JL, Poole RM (2001). Clinical importance of changes in chronic pain intensity measured on an 11-point numerical pain rating scale. Pain.

[8] Towheed TE, Maxwell L, Judd MG, Catton M, Hochberg MC, Wells G (2006). Acetaminophen for osteoarthritis. Cochrane Database Syst Rev.

[9] Maestri E, Formoso G, Giroldini R, Riccomi S, Magrini N, Marata AM (2010). I farmaci nel dolore persistente. Fibromialgia, osteoartrosi, mal di schiena. Pacchetti Informativi sui Farmaci.

[10] Holt WS, Mazzuca SA (1992). Prescribing behaviors of family physicians in the treatment of osteoarthritis. Fam Med.

[11] Luiza VL, Chaves LA, Silva RM, Emmerick IC, Chaves GC, Fonseca de Araújo SC (2015). Pharmaceutical policies: effects of cap and co-payment on rational use of medicines. Cochrane Database Syst Rev.

[12] Green CJ, Maclure M, Fortin PM, Ramsay CR, Aaserud M, Bardal S (2010). Pharmaceutical policies: effects of restrictions on reimbursement. Cochrane Database Syst Rev.

[13] Fretheim A, Håvelsrud K, MacLennan G, Kristoffersen DT, Oxman AD (2007). The effects of mandatory prescribing of thiazides for newly treated, uncomplicated hypertension: interrupted time-series analysis. PLoS Med.

[14] Bjerrum L, Larsen J, Kragstrup J (2001). Guidelines accompanied by changes in reimbursement rules. Effects on lipid-lowering drug prescribing. Scand J Prim Health Care.

[15] Jackevicius CA, Tu JV, Demers V, Melo M, Cox J, Rinfret S, Kalavrouziotis D, Johansen H, Behlouli H, Newman A, Pilote L (2008). Cardiovascular outcomes after a change in prescription policy for clopidogrel. N Engl J Med.

[16] Sakshaug S, Furu K, Karlstad Ø, Rønning M, Skurtveit S (2007). Switching statins in Norway after new reimbursement policy: a nationwide prescription study. Br J Clin Pharmacol.

[17] van Driel ML, Vander Stichele R, Elseviers M, De Sutter A, De Maeseneer J, Christiaens T (2008). Effects of an evidence report and policies lifting reimbursement restrictions for acid suppressants: analysis of the Belgian national database. Pharmacoepidemiol Drug Saf.

[18] Magrini N, Formoso G, Capelli O, Maestri E, Nonino F, Paltrinieri B (2014). Long term effectiveness on prescribing of two multifaceted educational interventions: results of two large scale randomized cluster trials. PLoS One.

[19] Magrini N, Formoso G, Marata AM, Capelli O, Maestri E, Voci C (2007). Randomised controlled trials for evaluating the prescribing impact of information meetings led by pharmacists and of new information formats, in general practice in Italy. BMC Health Serv Res.

[20] Sinnott SJ, Normand C, Byrne S, Woods N, Whelton H (2015). Copayments for prescription medicines on a public health insurance scheme in Ireland. Pharmaco Epidemiol Drug Saf.

[21] Twardella D, Brenner H (2007). Effects of practitioner education, practitioner payment and reimbursement of patients' drug costs on smoking cessation in primary care: a cluster randomised trial. Tob Control.

[22] Munson JC, Wahl PM, Daniel G, Kimmel SE, Hennessy S (2012). Factors associated with the initiation of proton pump inhibitors in corticosteroid users. Pharmacoepidemiol Drug Saf.

[23] National Collaborating Centre for Chronic Conditions (UK) (2008). Osteoarthritis. National Clinical Guideline for Care and Management in Adults.

[24] Kelly OB, Dillane C, Patchett SE, Harewood GC, Murray FE (2015). The inappropriate prescription of Oral proton pump inhibitors in the hospital setting: a prospective cross-sectional study. Dig Dis Sci.

[25] Moran N, Jones E, O'Toole A, Murray F (2014). The appropriateness of a proton pump inhibitor prescription. Ir Med J.

[26] Haastrup P, Paulsen MS, Begtrup LM, Hansen JM, Jarbøl DE (2014). Strategies for discontinuation of proton pump inhibitors: a systematic review. Fam Pract.

[27] Reeve E, Andrews JM, Wiese MD, Hendrix I, Roberts MS, Shakib S (2015). Feasibility of a patient-centered deprescribing process to reduce inappropriate use of proton pump inhibitors. Ann Pharmacother.

[28] Lapane KL, Hughes CM (2002). Optimising drug utilisation in long term care. Pharmacoeconomics..

[29] Labelle M, Beaulieu M, Paquette D, Fournier C, Bessette L, Choquette D (2004). An integrated approach to improving appropriate use of anti-inflammatory medication in the treatment of osteoarthritis in Québec (Canada): the CURATA model. Med Teach.

[30] Dreischulte T, Grant A, Donnan P, McCowan C, Davey P, Petrie D (2012). A cluster randomised stepped wedge trial to evaluate the effectiveness of a multifaceted information technology-based intervention in reducing high-risk prescribing of non-steroidal anti-inflammatory drugs and antiplatelets in primary medical care: the DQIP study protocol. Implement Sci.

