# Supplementary material for: A cluster randomized trial to measure the impact on nonsteroidal anti-inflammatory drug and proton pump inhibitor prescribing in Italy of distributing cost-free paracetamol to osteoarthritic patients
Source: BMC Fam Pract. 2019 Dec 6;20:169. doi: 10.1186/s12875-019-1050-4 (PMC6896368; doi:10.1186/s12875-019-1050-4)
Supplement: Supplementary file 1 — Additional file 1. questionnaire for GP. [file 12875_2019_1050_MOESM1_ESM.doc]

**QUESTIONANNAIRE FOR PHYSICIANS**

Dear Doctor,

We thank you for participating in our study on the use of paracetamol for patients with OA.

Now that we have completed the study, we ask you to please answer the few questions below regarding your and your patients’ adherence to and satisfaction with the study project.

The questionnaire is ANONYMOUS and can be returned directly to Dr. Alberto Gandolfi or to ……….

1. **Did you note any perplexity on your patients’ part regarding picking up the paracetamol at the Correggio Hospital Pharmacy?**

YES

NO

**1a) If so, for what reason?**

Trouble reaching the Correggio Hospital Pharmacy

Preference for picking up the drug at one’s usual pharmacy, even if paid for out-of-pocket

Preference for doing treatment with drugs other than paracetamol

Other

**2) Did your patients continue picking up the paracetamol throughout the entire treatment proposed?**

YES

NO

**2a) If not, for what reason?**

Ineffectiveness of drug

Intolerance to drug

Trouble reaching the Hospital Pharmacy

Opening hours of the Hospital Pharmacy

Other

**3) Your patients’ degree of satisfaction with the new modality of picking up paracetamol was overall:**

Excellent

Good

Modest

Low

Dissatisfied

**4) Your degree of satisfaction with the project was:**

Excellent

Good

Modest

Low

Dissatisfied

**5) Any suggestions or proposals you would make should the project be reproposed:**

**…………………………………………………………………………………………………………………………………………………………………**
